# CRISPR-Cas–associated SCC*mec* Variants in Methicillin-resistant *Staphylococcus aureus* Evade Rapid Diagnostic Detection

**DOI:** 10.1093/infdis/jiaf575

**Published:** 2025-11-19

**Authors:** Magdalena Podkowik, Alice Tillman, Courtney Takats, Heloise Carion, Gregory Putzel, Julian McWilliams, Benjamin See, Guiqing Wang, Sigridh Munoz-Gomez, Caitlin Otto, Karl Drlica, Luciano Marraffini, Alejandro Pironti, Sarah Hochman, Christopher Kerantzas, Bo Shopsin

**Affiliations:** Department of Microbiology, New York University Grossman School of Medicine, New York, New York, USA; Department of Medicine, Division of Infectious Diseases, New York University Grossman School of Medicine, New York, New York, USA; Antimicrobial-Resistant Pathogens Program, New York University Grossman School of Medicine, New York, New York, USA; Department of Microbiology, New York University Grossman School of Medicine, New York, New York, USA; Antimicrobial-Resistant Pathogens Program, New York University Grossman School of Medicine, New York, New York, USA; Department of Microbiology, New York University Grossman School of Medicine, New York, New York, USA; Antimicrobial-Resistant Pathogens Program, New York University Grossman School of Medicine, New York, New York, USA; Laboratory of Bacteriology, The Rockefeller University, New York, New York, USA; Howard Hughes Medical Institute, The Rockefeller University, New York, New York, USA; Department of Microbiology, New York University Grossman School of Medicine, New York, New York, USA; Antimicrobial-Resistant Pathogens Program, New York University Grossman School of Medicine, New York, New York, USA; Microbial Computational Genomic Core Lab, NYU Grossman School of Medicine, New York, New York, USA; Department of Microbiology, New York University Grossman School of Medicine, New York, New York, USA; Antimicrobial-Resistant Pathogens Program, New York University Grossman School of Medicine, New York, New York, USA; Department of Pathology, NYU Grossman School of Medicine, New York, New York, USA; Department of Pathology, NYU Grossman School of Medicine, New York, New York, USA; Department of Medicine, Division of Infectious Diseases, NYU Grossman Long Island School of Medicine, New York, New York, USA; Department of Pathology, NYU Grossman School of Medicine, New York, New York, USA; Public Health Research Institute, New Jersey Medical School, Rutgers University, Newark, NJ 07102, USA; Department of Microbiology, Biochemistry and Molecular Genetics, New Jersey Medical School, Rutgers University, Newark, New Jersey, USA; Laboratory of Bacteriology, The Rockefeller University, New York, New York, USA; Howard Hughes Medical Institute, The Rockefeller University, New York, New York, USA; Antimicrobial-Resistant Pathogens Program, New York University Grossman School of Medicine, New York, New York, USA; Microbial Computational Genomic Core Lab, NYU Grossman School of Medicine, New York, New York, USA; Department of Medicine, Division of Infectious Diseases, New York University Grossman School of Medicine, New York, New York, USA; Antimicrobial-Resistant Pathogens Program, New York University Grossman School of Medicine, New York, New York, USA; Department of Pathology, NYU Grossman School of Medicine, New York, New York, USA; Department of Microbiology, New York University Grossman School of Medicine, New York, New York, USA; Department of Medicine, Division of Infectious Diseases, New York University Grossman School of Medicine, New York, New York, USA; Antimicrobial-Resistant Pathogens Program, New York University Grossman School of Medicine, New York, New York, USA

**Keywords:** MRSA, SCC*mec*, CRISPR-Cas, rapid diagnostics

## Abstract

Rapid molecular assays guiding treatment of methicillin-resistant *Staphylococcus aureus* detect SCC*mec* (Xpert) or the SCC*mec*–*orfX* junction (BCID2). Sequence variation in this region can disrupt primer binding, yielding false-negative results. Investigation of a missed bloodstream infection linked escape to a CRISPR-Cas–associated SCC*mec* variant, leading to identification of 64 variants from 45 patients—2% of 2432 screened. Misdiagnosis was restricted to clonal complex 5, a hospital-associated lineage; 11 of 40 SCC*mec*/junctions evaded detection by BCID2 or Xpert. Variants had *mecA* instability and circulated in healthcare settings. Our findings reveal a unique escape mechanism and underscore a threat to diagnostic accuracy.

## Teaser

This study identifies methicillin-resistant *Staphylococcus aureus* clones carrying a CRISPR-Cas–linked SCC*mec* variant that escapes detection by rapid, point-of-care molecular diagnostics that are routinely used to guide antimicrobial therapy of bloodstream infections; the work has implications for clinical management.

Methicillin-resistant *Staphylococcus aureus* (MRSA) remains a leading cause of healthcare-associated infections worldwide. Although bloodstream infections decreased by approximately 13% between 2005 and 2012, progress has since plateaued [1]. Mortality remains high despite optimal therapy, making MRSA the primary driver of antimicrobial resistance-associated deaths globally [2].

Rapid PCR-based assays such as Xpert MRSA/SA Blood Culture (Cepheid) and Blood Culture Identification 2 (BCID2, BioFire) facilitate early MRSA detection and timely initiation of therapy that improve patient outcomes [3]. These assays detect *mecA* and species-specific markers (eg, *spa*), but rely on conserved sequences within *SCCmec* (Xpert) or the *SCCmec–orfX* junction (BCID2) to link *mecA* to *S. aureus* [4]. Sequence variation at this junction can disrupt primer binding, causing false negative assay results and impaired clinical decision-making [4, 5]. Knowing the prevalence and molecular basis of such MRSA variants is therefore critical to evaluating test performance.

Clustered regularly interspaced short palindromic repeats (CRISPRs) and associated Cas proteins mediate phage defense [6]. In MRSA, CRISPR-Cas systems occur within the SCC*mec* element and are likely acquired via horizontal gene transfer from coagulase-negative staphylococci (CoNS), given their high prevalence in CoNS (approximately 35%), but rarity in *S. aureus* (approximately 2%) [7]. Their impact on molecular MRSA detection is unknown.

We report a case of MRSA bacteremia in which resistance was undetected by rapid testing due to a CRISPR-Cas–associated *SCCmec* variant. Subsequent biobank screening revealed additional variants, including strains circulating in hospitals. Although currently infrequent, these variants compromise diagnostic accuracy, thus underscoring the need for genomic surveillance that shifts detection from reactive investigation of diagnostic failures to prospective monitoring of emerging diagnostic-escape variants as *S. aureus* evolves.

## METHODS

### Bacterial Isolates

The *S. aureus* isolate from the case patient was collected at NYU Langone Health's (NYULH) Long Island campus. The institutional biobank includes 9440 genome-sequenced isolates—5997 methicillin-susceptible *S. aureus* (MSSA) and 3443 MRSA—from 6670 patients over 2 years (March 2022−March 2024). Biobank isolates originated from NYULH Brooklyn (*n* = 2773) and Manhattan (*n* = 6667); Long Island isolates are not included. MRSA isolates were obtained from clinical and routine admission screening cultures, and MSSA were from blood and screening cultures only; all were handled identically before sequencing. MRSA was identified using Vitek 2 (BioMérieux, France) for clinical cultures and CHROMagar MRSA II (BD Diagnostics, NJ) for screening cultures. The study was approved by the NYULH Institutional Review Board (s24-01872).

### Genomic Analysis

**Figure 1.**
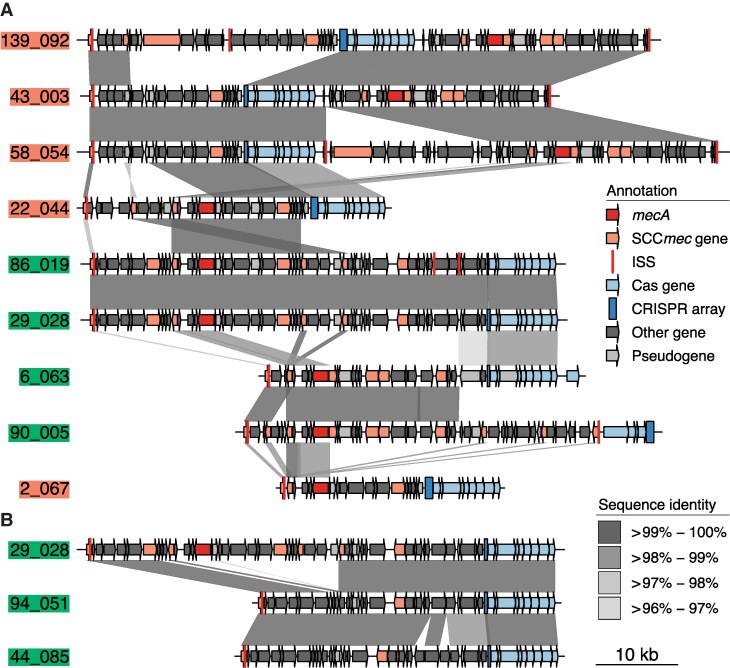
Structural comparison of SCC*mec*-associated III-A CRISPR-Cas elements in *S. aureus*. *A*, Comparison of six representative CRISPR-Cas elements from *mecA*-positive strains (90_005, 2_067, 6_063, 139_092 [index case], 22_044, 29_028), and 3 related clones (58_054, 43_003, 86_019). Subclones and related clones were selected based on SCC*mec* type, clonal complex, CRISPR-Cas subtype, and associated phage defense gene profiles (DefenseGroups *A*–*F*; Supplementary Table 1 and Supplementary Methods). Elements are ordered by overall similarity; gray shading highlights conserved regions. Annotations: SCC*mec* components (orange); CRISPR-Cas genes (blue); *mecA* (red); and SCC*mec*–orfX junction (red bars). Genomic regions spanning *orfX* to the CRISPR-Cas locus were defined by positional features, compared pairwise (blastn, default settings), and visualized with genoPlotR v0.8.11 (bit score <10 excluded). Junction sites and duplications were annotated via tblastn (query: GenBank ADC79452.1; PGAP CDS as subject; ≥95% query coverage) and blastn of the final 15 nt of *orfX* queried against the assembly (≥80% identity, 100% query coverage, word size 5, *e* value ≤100). Strains predicted in silico to evade detection [9] by SCC*mec*–*orfX* primer sets [10] are color coded (green, detectable; scarlet, undetectable; see also Supplementary Table 1). These predictions matched empirical SCC*mec*/junction detection by commercial Xpert and BCID2 assays, with one exception: the element from strain 2_067 were detected by both assays despite being undetectable by research primers, highlighting variability in primer performance. *B*, Comparison of *mecA*-negative strains 94_051 and 44_058 with their closest *mecA*-positive match from panel (*A*) (29_028). Labels, color schemes, and annotation conventions as in panel (*A*). Presence of *pls* and *cstB/R*, along with phylogenetic context (Figure 2), suggests *mecA* loss from an ancestral MRSA background.

Genomic DNA was extracted using MagMax DNA Multisample 2.0 (Thermo Fisher Scientific, Waltham, MA). All strains except 139_092 were sequenced and assembled using Illumina [8] (coverage 120–520x; N50 = 46–847 kbp). A subset of strains (Figure 1) underwent additional Nanopore sequencing (76–104x; Plasmidsaurus, Eugene, OR); assemblies were polished with Illumina reads using *pypolca* v0.3.1 (“–careful” mode; https://github.com/gbouras13/pypolca), yielding hybrid assemblies. Strain 139_092 underwent both Nanopore and Illumina sequencing and assembly by Plasmidsaurus. All hybrid assemblies yielded closed chromosomes. Details of genome annotation, typing, phylogenetic analysis, and transmission events are provided in Supplementary Methods.

### Rapid Diagnostic Assays

Detectability of MRSA isolates carrying CRISPR-Cas SCC*mec* elements used BCID2 (bioMérieux, France) and Xpert (Cepheid, Sunnyvale, CA) assays. Tests were performed blinded according to manufacturers’ protocols, using a 0.5 McFarland suspension of *S. aureus* colonies in Vitek sterile saline (bioMérieux, France), diluted 10^4^-fold before inoculation.

## RESULTS

### Case

An 88-year-old man developed nosocomial bacteremia at NYULH-Long Island. Blood cultures grew gram-positive cocci in clusters, and the BCID2 assay reported *S. aureus* “Detected” but “Not Detected” for the “*mecA*/*C* and MREJ (*mec* right extremity junction)” target used to assess the presence of the SCC*mec*–*orfX* junction. Based on this result, empiric vancomycin was de-escalated to oxacillin for approximately 48 hours. When culture and susceptibility testing later confirmed MRSA, therapy was switched to daptomycin plus ceftaroline for 6 weeks for treatment of complicated bacteremia, leading to clinical resolution.

### Genomic Analysis

This case prompted investigation into the probability of false-negative BCID2 results at our hospitals, beginning with genome sequencing of the index isolate. The strain (139_092; Supplementary Table 1), a clonal complex 5 (CC5) hospital-associated MRSA, harbored *mecA* but lacked typical SCC*mec* type II or IV cassettes associated with CC5 [11]. Instead, it contained a variant SCC*mec* element encoding a Class 1 Type IIIA CRISPR-Cas system (Figure 1*A*).

Methicillin-resistant coagulase-negative staphylococci (MR-CoNS) frequently carry SCC*mec*, serving as both a source and a reservoir for SCC*mec* in *S. aureus* [7]. To avoid false positive results when MR-CoNS and MSSA coexist in a sample, some rapid MRSA assays, such as BCID2, do not exclusively target *mecA*. Instead, they also detect the SCC*mec*-*orfX* integration site to specifically link *mecA* to *S. aureus*. Sequence analysis revealed that the CRISPR-Cas–associated SCC*mec* element contained alterations at the *orfX*-SCC*mec* junction that likely prevent detection by standard assay primers (Figure 1*A*).

### Prevalence of CRISPR-Cas SCCmec Elements

**Figure 2.**
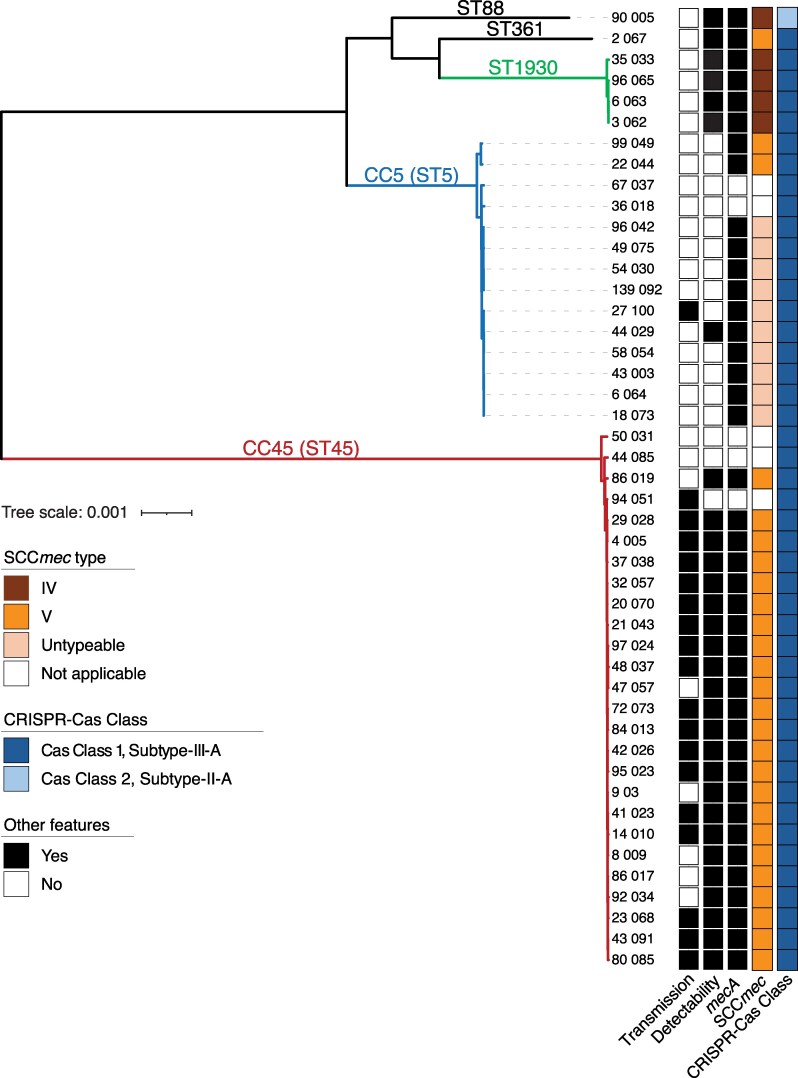
Maximum likelihood phylogeny of isolates carrying SCC*mec*-associated III-A CRISPR-Cas elements. The phylogeny includes one representative isolate per patient (*n* = 46), except for one patient from whom two distinct variants were recovered (43_003 and 44_029; see Supplementary Table 1). Sequence type (ST) and clonal complex (CC) were assigned by multilocus sequence typing; CCs are indicated when the ST is the recognized founder of that clonal complex (eg, CC5 for ST5). Colored blocks denote CC, putative transmission events (<20 SNPs with epidemiological link), predicted detectability by Xpert and BCID2, *mecA* status, SCC*mec* type, and CRISPR-Cas Class (light blue, Class 2; dark blue, Class 3). Scale bars indicate substitutions per site. Core genome size: 2 345 248 bp; variable sites 65 220 bp.

Finding a CRISPR-Cas–associated SCC*mec* variant as the likely cause of diagnostic failure in our index case prompted an investigation into whether similar undetectable elements are circulating more broadly. To assess their prevalence—and their potential impact on the performance of rapid diagnostic assays—we screened our biobank of >9000 genome sequences of *S. aureus* isolates from NYULH's Manhattan and Brooklyn campuses for CRISPR-Cas systems. We identified 5 MSSA and 59 MRSA isolates from 5 and 40 patients, respectively, carrying CRISPR-Cas systems. Among 41 unique MRSA strains (one per patient, except for one patient from whom two distinct variants were recovered), we identified 39 SCC*mec*-associated CRISPR-Cas Class 1, Type IIIA systems and one Class 2 system—the latter, to our knowledge, the first reported in *S. aureus* (Supplementary Table 1). Six variant subclones (DefenseGroups A–F; Supplementary Table 1, Methods, and Figure 1*A*) were defined based on unique combinations of Clonal Complex, SCC*mec* type, CRISPR-Cas subtype, and DefenseCombinationID—the last reflecting the arrangement of associated antiphage defense genes. Among the five MSSA strains, at least three (94_051, 44_058, and 36_018) retained presumptive residual SCC*mec* genes (eg, *cstB/R*; Figure 1*B*). Together with sequence homology and phylogenetic context (Figure 2), these features suggest *mecA* loss. Although SCC*mec* excision has been described as a mechanism for *mecA* loss in MRSA, its occurrence in CRISPR-Cas–associated SCC*mec* elements is novel and raises the possibility that these elements destabilize *mecA*, a hypothesis warranting further study given the mutagenic potential of type III-A CRISPR-Cas systems in staphylococci [12].

CRISPR-Cas systems were concentrated primarily in CC5 (14 strains and 13 patients) and CC45 (26 strains and 26 patients) (Figure 2 and Supplementary Table 1). Each clonal complex was associated largely with a specific phage defense gene profile. This pattern, supported by phylogenetic analysis (Figure 2), suggests multiple independent acquisitions followed by vertical inheritance with limited horizontal transfer.

### Impact on Diagnostic Assay Performance

When we tested representative isolates of the six *mecA-*positive CRISPR-Cas subclones (DefenseGroups A–F; Supplementary Table 1 and Supplementary Methods), we found that diagnostic escape was restricted to CC5, a dominant hospital-associated lineage [11]: the two CC5 subclones had SCC*mec* or junction sites undetectable by Xpert and BCID2 assays, respectively. Based on replicate results with related clones (Supplementary Table 1 and Supplementary Methods), we estimate that 11 of the 40 SCC*mec*/junctions in *mecA*-positive isolates would fail detection by at least one platform, while 31 would be identified. As with our case patient, test failures were associated with polymorphisms in SCC*mec* or the junction region that likely disrupt assay recognition by preventing primer binding (Figure 1*A*).

Notably, the Xpert assay yielded results of “MRSA Positive/SA Positive” even without detection of SCC*mec*, indicating that its rules-based algorithm conditions were met for *mecA* and *spa* cycle threshold (Ct) values (Supplementary Table 1). As a result, the Xpert system may be more robust than BCID2 for detecting certain CRISPR-Cas–associated *SCCmec* MRSA variants. However, the apparent advantage of the Xpert system may reflect our controlled conditions and bacterial inoculum; head-to-head evaluation in clinical blood cultures and real-world settings is needed to determine whether this performance difference holds in practice.

### Healthcare Transmission and Geographic Distribution

CRISPR-Cas SCC*mec*–containing *S. aureus* strains were identified predominantly in patients residing in Brooklyn (29 out of 45), including most CC45 clones (22 out of 26) Supplementary Table 1). Many affected patients had a history of prior hospitalization and/or healthcare exposure (29 of 45), particularly in long-term care facilities, where 20 strains were isolated from residents. Clustering by healthcare exposure and geography, coupled with restricted clonality, suggests that CRISPR-Cas SCC*mec* variants are circulating in healthcare settings among highly susceptible hosts.

To assess transmission, we applied spatial-temporal overlap criteria [13], identifying a genomically and epidemiologically linked cluster of 12 patients (direct transmissions, Supplementary Table 1). Eleven resided in Brooklyn; nine transmissions occurred at NYULH Brooklyn and three at NYULH Manhattan, consistent with intranetwork spread. All cases involved CC45, and all patients lived in long-term care facilities or the same senior apartment complex. Using relaxed criteria—allowing acquisition after the index case had left the ward [13] (Indirect transmissions, Supplementary Table 1)—we identified additional CC45 events and a CC5 cluster involving nondetectable strains. Recovery of near-identical isolates from linked hosts supports nosocomial spread, including secondary transmission.

### Screening Public Genomes for CRISPR-associated SCC*mec* Variants

Screening 70597 *mecA*-positive MRSA assemblies from GenBank identified *cas* systems in 1.4% (*n* = 1000; nearly all Class 1, subtype IIIA [*n* = 978]), a percentage similar to our biobank. Forty-seven assemblies met our stringent *mecA*-associated CRISPR-Cas colocalization criterion (Supplementary Methods), representing a conservative lower bound of high-confidence analogs for our NYULH variants (Supplementary Table 2). Additional examples are likely masked by fragmented assemblies. These isolates span diverse sequence types, including CC5 and CC45, and geographic regions, suggesting independent acquisitions. In silico primer analysis predicted that 24 (51%) would evade detection. Thus, SCC*mec*-linked CRISPR systems capable of diagnostic escape are widely distributed.

## DISCUSSION

Our findings indicate that evolution of MRSA can yield variants that evade widely used rapid molecular diagnostics. We identified CRISPR-Cas–associated SCC*mec* elements, including the first Class 2 CRISPR-Cas system reported in *S. aureus*, that disrupt a key target of current assays rendering certain affected strains undetectable. This mechanism of diagnostic escape is distinct from novel *mec* homologs and small-scale SCC*mec*/*orfX* junction polymorphisms. Phylogenetic analysis suggests multiple independent emergences, now concentrated in CC45 and CC5, with evidence of ongoing circulation in healthcare settings. That diagnostic escape is concentrated in CC5—the dominant hospital-associated lineage [11 ]—is particularly concerning, since it suggests that the most clinically relevant clone may become the hardest to detect. Continued spread, potentially accelerated by selective pressure from surveillance and control measures [14], for example targeted screening and decolonization that treats only detectable strains, could compromise the reliability of rapid MRSA diagnostics guiding early therapy.

These results highlight a broader challenge: the continual threat that pathogen evolution places on diagnostic accuracy. The clinical consequences of escape variants depend on local prevalence and the extent to which rapid test results influence antibiotic selection. While missed detection of MRSA colonization may hinder de-escalation and infection control, a false-negative in bloodstream infection risks delaying definitive effective therapy. Local assessment of escape variant frequency is therefore essential, both to refine current detection methods and to inform the design of alternative testing platforms. In settings where such variants are circulating, clinicians should interpret negative rapid MRSA results in invasive infections with caution.

Early recognition of such variants depends on the vigilance of clinical laboratories and front-line physicians, who are often the first to encounter diagnostic anomalies. However, colonizing and most nonbloodstream MRSA isolates are not routinely subjected to molecular testing due to cost and logistical complexity. Consequently, variants can circulate undetected. Outside our index case, only 4 of 40 patients harboring potentially undetectable MRSA variants had isolates evaluated by PCR-based blood culture systems—one by BCID2, which did not report resistance markers, and three via Xpert, which did. These findings illustrate how easily such variants can evade routine surveillance and spread silently within healthcare settings.

In summary, our results underscore the need for genomic surveillance [15] and in silico primer screening [14] to detect diagnostic-escape variants before they become widespread. Ongoing monitoring for new variants and collaboration with diagnostic manufacturers to update molecular targets are essential to preserve early MRSA detection.

## Supplementary Material

jiaf575_Supplementary_Data

## Supplementary Data

Supplementary materials are available at *The Journal of Infectious Diseases* online (http://jid.oxfordjournals.org/). Supplementary materials consist of data provided by the author that are published to benefit the reader. The posted materials are not copyedited. The contents of all supplementary data are the sole responsibility of the authors. Questions or messages regarding errors should be addressed to the author.
