# Supplementary material for: CRISPR-Cas–associated SCCmec Variants in Methicillin-resistant Staphylococcus aureus Evade Rapid Diagnostic Detection
Source: J Infect Dis. 2025 Nov 19;233(2):e534–40. doi: 10.1093/infdis/jiaf575 (PMC13017219; doi:10.1093/infdis/jiaf575)
Supplement: jiaf575_Supplementary_Data [file jiaf575_supplementary_data.zip › supplemental_materials_revised_v4.1.docx]

**Supplementary Information For**

**CRISPR-Cas-associated SCC*mec* variants in methicillin-resistant *Staphylococcus aureus* evade detection by rapid diagnostics**

Magdalena Podkowik,^a,b,c^  Alice Tillman, ^a,c^ Courtney Takats,^a,c^ Heloise Carion,^d,e^ Gregory Putzel,^a,c,f^, Julian McWilliams ^a,c^, Benjamin See,^g^ Guiqing Wang,^g^ Sigridh Munoz-Gomez,^h^ Caitlin Otto,^g^ Karl Drlica,^I,j^ Luciano Marraffini,^d,e^ Alejandro Pironti,^c,f^ Sarah Hochman,^b,c^ Christopher Kerantzas,^g^ Bo Shopsin^a,b,c,#^

^a^Department of Microbiology, New York University Grossman School of Medicine, New York, NY 10016, USA;

^b^Department of Medicine, Division of Infectious Diseases, New York University Grossman School of Medicine, New York, NY 10016, USA;

^c^Antimicrobial-Resistant Pathogens Program, New York University Grossman School of Medicine, New York, NY 10016, USA;

^d^Laboratory of Bacteriology, The Rockefeller University, New York, NY 10065, USA;

^e^Howard Hughes Medical Institute, The Rockefeller University, New York, NY 10065, USA;

^f^Microbial Computational Genomic Core Lab, NYU Grossman School of Medicine, New York, NY 10016, USA;

^g^Department of Pathology, NYU Grossman School of Medicine, New York, NY 10016, USA;

^h^Department of Medicine, Division of Infectious Diseases, NYU Grossman Long Island School of Medicine, New York, NY 11501, USA;

^i^Public Health Research Institute, New Jersey Medical School, Rutgers University, Newark, NJ 07102, USA;

^j^Department of Microbiology, Biochemistry & Molecular Genetics, New Jersey Medical School, Rutgers University, Newark, NJ 07102, USA.

**This PDF file includes:**

**Supplementary Information text (Methods)**

**References for SI citations**

**Supplementary Information Text (Methods)**

**Genome annotation, typing, and phylogenetic analysis**

Long-read assemblies were annotated with PGAP v2024-07-18.build 7555 (https://github.com/ncbi/pgap), SCCmecFinder v1.2.0 (<https://github.com/> rpetit3/sccmec), and CRISPRCasFinder v4.3.2 (https://doi.org/10.1038/s41467-022-30269-9). DefenseFinder v2.0.0 (<https://doi.org/10.1038/s41467-022-30269-9>) was applied to Illumina-based genome assemblies and to PGAP-predicted proteomes derived from long-read assemblies. Clonal complexes were assigned using MLST v. 2.23.0 (https://github. com/tseemann/mlst). SNVs, core genome alignments, and phylogenies (visualized with iToL) were generated with Snippy v4.6.0 (<https://github.com/tseemann/snippy>) using the long-read assembly of strain 139_092 as reference (1).

**Transmission events**

Transmission among patients for the 46 CRISPR-Cas*–*carrying isolates (from 45 patients) was estimated using a stringent threshold of 20 single-nucleotide polymorphisms between patients (2). Isolates meeting this genetic similarity criterion were analyzed for clustering. Clusters were defined as two or more patients with direct ward contact (same units [excluding the emergency department], room, procedure, or providers, with overlapping admission dates) or indirect ward contact (same ward, overlapping admission dates within 6 months) (2), using temporal and spatial electronic record data to confirm contacts.

**DefenseFinder types and their distribution among biobank clones**Defense systems were identified using DefenseFinder v2.0.0 (3)(Methods), and each isolate was assigned a DefenseCombination ID based on the alphabetical set of systems detected (e.g., ID #15: AbiD; CAS_Class1-Subtype-III-A; FS_Sma; FS_Sma; gcu233; PD-T4-9; Supplementary Table 1). To allow broader classification, isolates were grouped into six DefenseGroups (A–F) based on assessment of shared motifs and gene organization. Although less rigid than ID definitions, these groupings were guided by parsimony—the assumption that accumulated differences reflect relatedness and similar patterns suggest shared ancestry. Consistent with this framework, each DefenseCombination ID mapped to a single clonal complex, suggesting that clonal lineage precedes diversification in antiphage defense composition. This supports the use of parsimony-based grouping, as variation appears to accumulate within lineages rather than through frequent horizontal exchange.

DefenseGroups also correlated with detection profiles, as reflected by boldfaced isolates in Supplementary Table 1. For example, group D (ID types 6, 14, 15) and group E (types 18, 20), despite carrying distinct IDs, showed identical detection patterns. This congruence support the use of DefenseGroups distinguish between detectable and undetectable variants among isolates with shared or closely related clonal complexes and SCC*mec* types.

**Screening public genomes for CRISPR-associated SCC*mec* variants**

To estimate the prevalence of CRISPR-associated SCC*mec* variants beyond New York City, we screened publicly available *S. aureus* genomes in GenBank. Defense systems were identified from 115,924 *S. aureus* genome assemblies in the NCBI database (<https://www.ncbi.nlm.nih.gov/>; accessed September 17, 2025), including 18,949 from RefSeq, (curated reference-quality), and 96,976 from large multi-isolate sequencing projects.

The geographic location of isolates was obtained by cross-referencing BioSample identifiers with Entrez Direct v.24.0 ([https://www.ncbi.nlm.nih.gov/books/NBK179288/](https://www.ncbi.nlm.nih.gov/books/NBK179288/?utm_source=chatgpt.com)). Sequence types were assigned using mlst v.2.19.0 (<https://github.com/tseemann/mlst>). Defense systems were annotated using DefenseFinder v.2.0.0 (<https://doi.org/10.1038/s41467-022-30269-9>), and the presence of *mecA* (NCBI ID: NC_007793.1, SAUSA300_0032) and *orfX* (SAUSA300_0026) was determined using blastn v. 2.11.0 (4) (≥90% identity, ≥80% coverage). Among *mecA-*positive assemblies (*n* = 70,597), assembly levels included contig (*n* = 60,103), scaffold (*n* = 9,062), complete genome (*n* = 1,344), and chromosome (*n* = 88).

To extend observations from NYULH, isolates showing *mecA*-associated CRISPR-Cas systems within SCC*mec* elements that escape PCR detection, we screened public assemblies for analogous configurations in which *cas* genes within SCC*mec* occur near *mecA*. Because complete *S. aureus* genomes are rare and *SCCmec* regions are frequently fragmented and split across multiple contigs, it was not possible to reconstruct neighboring gene relationships. Instead, we applied a stringent, specificity-focused approach, in which we restricted analysis to assemblies in which *mecA* and CRISPR-Cas loci were present on the same contig.

The 70,597 *mecA*-positive assemblies were analyzed with CRISPRCasFinder v4.3.2 (5) to identify *cas* gene clusters and CRISPR arrays. Arrays located ≤200 nt from a *cas* cluster were considered part of the same CRISPR-Cas system, consistent with our closed NYULH assemblies. Assemblies were retained only when *mecA* and a CRISPR-Cas system occurred on the same contig. The *mecA*–CRISPR-Cas distance, defined as the minimal interval between the *mecA* midpoint and the start or end of the *cas* cluster, had a median of 14,536 nt (IQR: 14,218–18,670 nt). The resulting set (47 assemblies; Supplementary Table 1) was enriched for scaffold and complete-genome assemblies (22/47 [47%] vs 8% in the total dataset), reflecting the greater contiguity required for detection.

The 47 assemblies were evaluated in silico using PCRInSilico v0.1.0 (6) to predict amplicon length by standard MRSA detection primer sets (7) and to estimate the potential for diagnostic escape.

**References for SI citations**

1. A. St John *et al.*, Capsular Polysaccharide Is Essential for the Virulence of the Antimicrobial-Resistant Pathogen *Enterobacter hormaechei*. *mBio* **14**, e0259022 (2023).

2. F. Coll *et al.*, Definition of a genetic relatedness cutoff to exclude recent transmission of meticillin-resistant *Staphylococcus aureus*: a genomic epidemiology analysis. *Lancet Microbe* **1**, e328-e335 (2020).

3. F. Tesson *et al.*, Systematic and quantitative view of the antiviral arsenal of prokaryotes. *Nature communications* **13**, 2561 (2022).

4. S. F. Altschul, W. Gish, W. Miller, E. W. Myers, D. J. Lipman, Basic local alignment search tool. *J Mol Biol* **215**, 403-410 (1990).

5. D. Couvin *et al.*, CRISPRCasFinder, an update of CRISRFinder, includes a portable version, enhanced performance and integrates search for Cas proteins. *Nucleic Acids Res* **46**, W246-W251 (2018).

6. J. D. Limberis, J. Z. Metcalfe, primerJinn: a tool for rationally designing multiplex PCR primer sets for amplicon sequencing and performing in silico PCR. *BMC Bioinformatics* **24**, 468 (2023).

7. A. Huletsky *et al.*, New real-time PCR assay for rapid detection of methicillin-resistant *Staphylococcus aureus* directly from specimens containing a mixture of staphylococci. *J Clin Microbiol* **42**, 1875-1884 (2004).
